# Thermal Ablation and Rapid On‐Site Cytology for Lung Nodules—A ‘One‐Stop Shop’ Approach for Small Lesions: Experience in 8 Patients

**DOI:** 10.1111/1754-9485.70072

**Published:** 2026-02-11

**Authors:** Andrei B. Gorgos, Damien Olivié, Philippe Stephenson, Edith Filion, Houda Bahig

**Affiliations:** ^1^ University of Montreal Montreal Quebec Canada

## Abstract

**Aim:**

This technical report describes our experience with eight patients with small lung lesions (less than 2 cm) treated by thermal ablation after rapid on‐site cytological confirmation of cancer diagnosis.

**Materials and Methods:**

Six patients were treated by microwave ablation and two by cryoablation, under local anaesthesia. Small, easily accessible tumours with low *a priori* risk of complications were selected. Serial thoracic CT scans were performed to assess for local recurrence, and overall survival was recorded.

**Results:**

Complete ablation under local anaesthesia was possible in all patients with no major complications; all patients were discharged on the same day. No hospital admissions were recorded within 30 days after the procedures, and no local recurrences were identified after an average follow‐up of 40 months (28–60 month range). Six patients are still alive, whereas two died of causes unrelated to the initial thermoablation treatment.

**Conclusion:**

We describe a favourable outcome of carefully selected patients who benefit from a rapid diagnosis‐treatment sequence under local anaesthesia. Microwave ablation is favoured, in our experience, over cryoablation for this patient population due to shorter treatment time.

**Abbreviations**
SABRT: stereotactic ablative radiation therapy
TA: Thermal Ablation

## 1 Introduction

Thermal ablation (TA) treatment of lung cancer is a technique currently available in many medical centres across the world, mainly as an alternative treatment for non‐surgical candidates [1]. In such patients, no randomised controlled prospective study exists that specifically compares outcomes for local recurrence and overall mortality between treatment by sublobar surgical resection, stereotactic ablative radiation therapy (SABRT), and thermal ablation. However, limited available data demonstrate good outcomes of TA performed on lung lesions inferior to 2 cm in diameter, as opposed to larger lesions, which compare favourably in terms of overall survival and local recurrence to general SABRT and sublobar resection figures []. Various TA techniques have been described in the literature; lung lesion treatment at our centre utilises mainly microwave ablation and cryoablation.

For patients with inoperable disease (non‐small cell lung cancer or oligometastatic disease), general clinical practice most often consists of percutaneous biopsy of suspicious lung lesions, followed by subsequent TA treatment performed on a separate day versus SABRT treatment (typically 1–8 sessions). The purpose of this brief report is to describe our institutional experience with a rapid treatment sequence, in which diagnosis and treatment occur during the same session. To this effect, patients referred for suspicious lung nodules present to the radiology suite the morning of the procedure and undergo a CT‐guided transthoracic biopsy/rapid on‐site cytology diagnosis, immediately followed by thermal ablation under local anaesthesia. Same day discharge is anticipated, following a relatively short surveillance period.

## 2 Materials and Methods

### 2.1 Patients

An Institutional Review Board approval was obtained for this retrospective analysis, and informed consent was waived. A multidisciplinary team comprising radiologists, radiation oncologists, oncologists, respirologists, and thoracic surgeons carefully selected non‐operable patients suitable for the same session diagnosis and treatment procedure, using the following criteria: lung nodules less than 2 cm in size (suspected primary neoplasia or oligometastatic disease), localised more than 1 cm from the pleura, non‐fissural, non‐central location (more than 2 cm from the hila and major vascular structures), and absence of gross bullous emphysematous change in the treatment area. For metastatic disease, only solitary lesions were selected, whereas patients with progression of the primary tumour within the 3 months prior to the TA were excluded. Once the case was deemed suitable, based on imaging (CT appearance and evolution and/or PET scan radiotracer uptake), patients were contacted, and the nature of the intervention, risks, and potential benefits were discussed at length. A total of eight patients were treated, between February 2020 and October 2022.

### 2.2 Procedure

**FIGURE 1:**
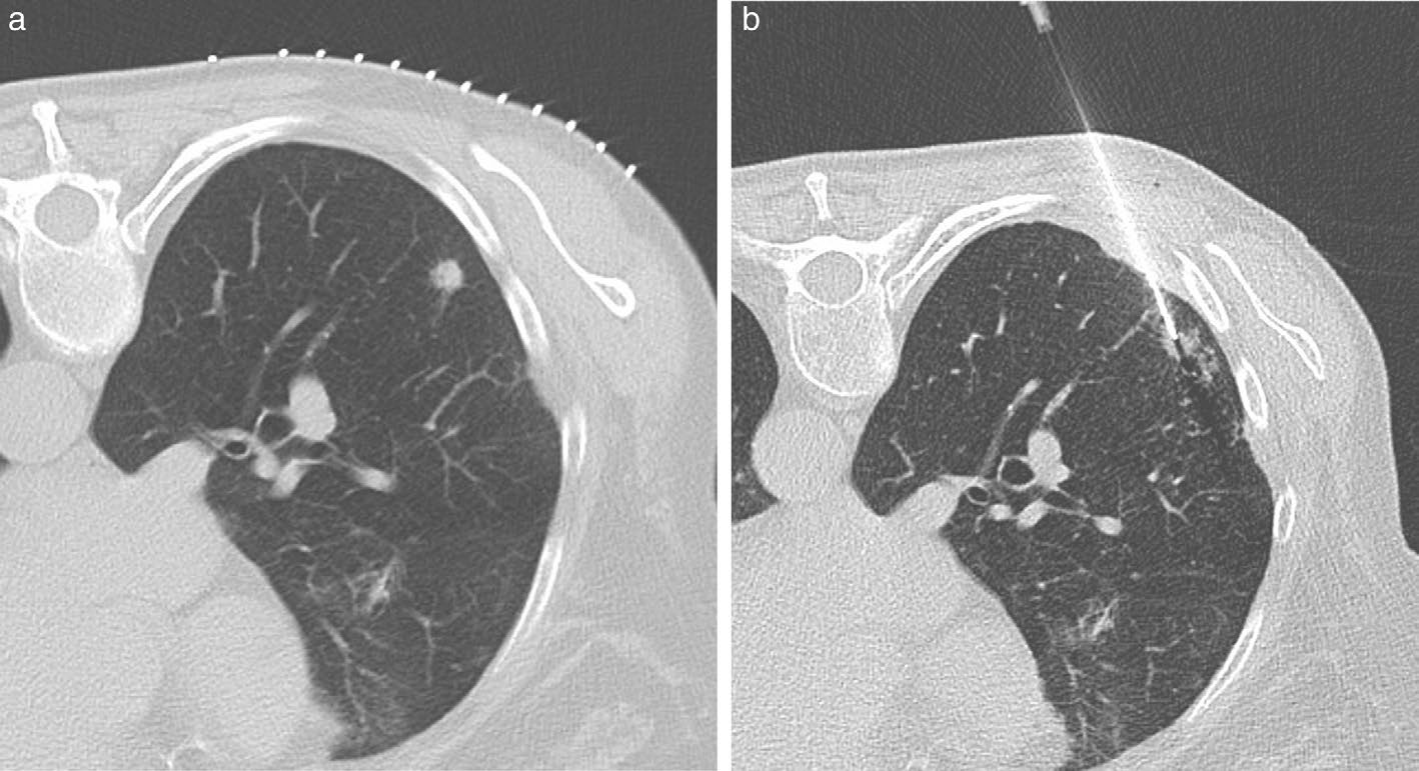
(a) Small right lower lobe solid nodule suspicious for primary lung neoplasia, in a 73‐year‐old female patient. (b) Transthoracic biopsy under local anaesthesia, followed by rapid on‐site cytological confirmation of cancer by the pathologist.

**FIGURE 2:**
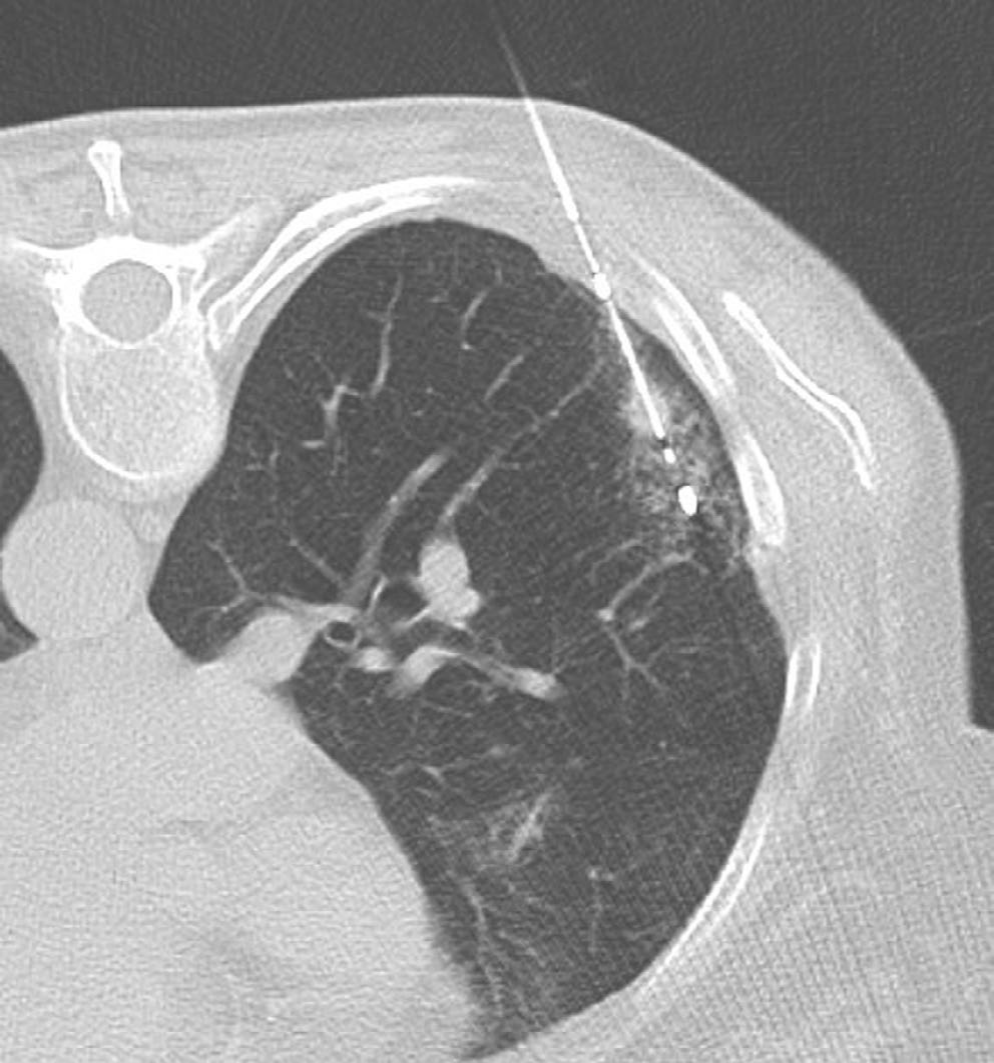
Microwave ablation needle placement through the lesion.

**FIGURE 3:**
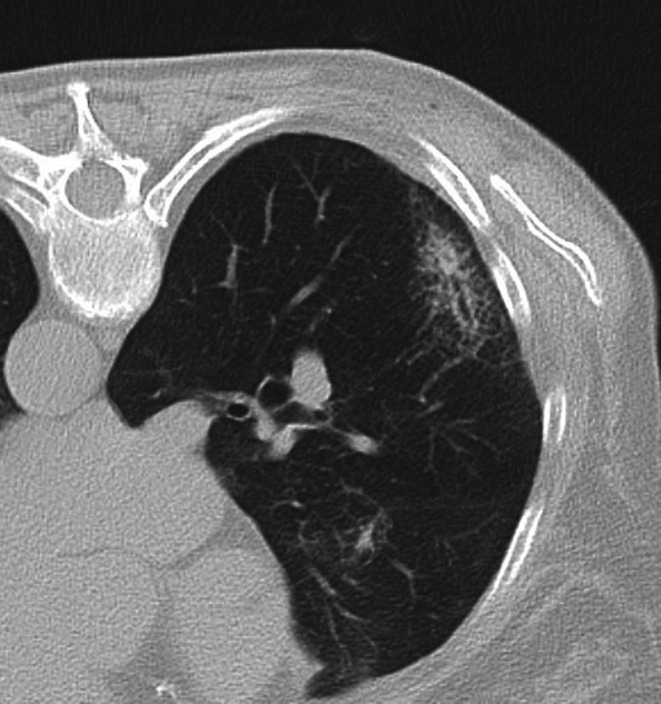
Post‐treatment imaging shows expected ground glass opacity and consolidation, representing an area of haemorrhage and coagulation necrosis.

Two radiologists with 15‐year experience performed all procedures. An aseptic technique was used to perform a thorough local pleural anaesthesia, under CT guidance. The standardised method of pleural anaesthesia used was described in detail in another paper [2]. Briefly, skin anaesthesia is performed with a small quantity of local anaesthetic (xylocaine 1%–2%). Subsequently, a 19 G coaxial needle (Temno Evolution, Merit Medical Ireland Ltd., Parkmore Business Park West, Galway, Ireland) was placed into the extra‐pleural space, between the extra‐thoracic fascia and the parietal pleura. At this point, approximately 10 cc of local anaesthetic was injected, in order to achieve proper pleural anaesthesia, and the coaxial needle was advanced to the target nodule, in order to perform the cytological analysis, via a 25 G biopsy needle (Chiba, Cook Incorporated, Bloomington, IN). (Figure 1a,b). An experienced pathologist was available to analyse the specimen and determine the presence or absence of neoplasia, by rapid cytological analysis. The pathologist accompanied by a technician was called to the radiology suite at the beginning of the intervention (which allowed them approximately 15 min to arrive with a microscope stand). The specimens were obtained and analysed (a diagnosis was confidently reached within four attempts). In the first four cases, a Cyberknife fiducial was inserted prior to TA needle placement in proximity of the lesion, in case significant haemorrhage occurred, which might have obscured the nodule and prevented accurate needle placement‐since no significant haemorrhage occurred and one case of tumoral seeding onto the Cybernknife fiducial was recorded; this practice was stopped for the remaining cases. The coaxial needle was removed, and the TA probe was inserted, following the same path as the co‐axial needle; treatment occurred according to the manufacturer protocol (Figure 2). Either a 14 G microwave ablation needle (Emprint, Covidien, Mansfield, MA) or a 14 G cryoablation needle (Ice Force, Boston Scientific, Yokneam, Israel) were used. Within each treatment volume, we included the lesion as well as a minimum of 1‐cm additional healthy lung tissue margin (Figure 3). Whereas for these patients microwave ablation is preferred, mainly because of its rapid treatment protocol (2–5 min generally), two patients out of the eight were treated by cryoablation (due to a temporary unavailability of the microwave ablation device). The ablation technique specifics are not part of this manuscript. Patients were observed in the radiology unit for 3–5 h post‐procedure; sequential x‐rays were performed for all patients approximately 1 h and 3 h post‐procedure, to assess for the development of pneumothorax.

### 2.3 Follow‐Up

A baseline CT without contrast was obtained at the end of the intervention, and another chest CT was scheduled in 6–8 weeks. Subsequent follow‐up was left at the charge of the referring clinician (thoracic surgeon or radiation oncologist), generally planned for 3–6‐month intervals. Local recurrence was defined as increasing density diameter and/or progressive bulging margins on serial CT‐scans subsequent to the first post‐TA imaging (at 6–8 weeks) (PET‐CT and contrast enhancement were not consistently used for this purpose).

## 3 Results

A total of eight patients were selected and scheduled for the rapid on‐site cytology/TA procedure, including 5 males and 3 females, ages 51 to 71 years old. On‐site cytological confirmation of neoplasia was possible in all cases, prior to TA. Of the eight patients, primary lung cancer (non‐small cell) and 6 metastatic nodules were cytologically confirmed (1 melanoma, 2 gastrointestinal, 2 oropharyngeal, and 1 breast primary neoplasias), 4 in the upper lobes, 4 in the lower lobes, ranging from 7 mm–11 mm (average size 9 mm). Seven nodules were solid, and one was subsolid. Six patients out of 8 had positive PET‐scans within the 3 months prior to the intervention.

All patients were successfully treated under local anaesthesia. One patient received light conscious sedation (50 micrograms of Fentanyl, 1 mg Versed) in addition to local anaesthetic; all other patients were treated with local anaesthesia only, with minimal discomfort. One patient required drainage of a 15 mm pneumothorax using a Seldinger technique and a needle path parallel and in close proximity to the initial co‐axial needle, thus taking advantage of the pleural anaesthesia already in effect in order to minimise pain. The pigtail was removed 4 h later, the same day. One patient developed a small, 4‐mm pneumothorax that required no drainage. The remaining six patients did not have any significant pneumothorax. No significant haemorrhage, haemoptysis, or other complications were observed in any of the patients.

After the intervention, patients were observed in the radiology nursing area for a total of 3 h–5 h. All patients were discharged the same day.

No patient required hospital admission within the 30 days following the intervention. At the time of writing this paper, 6 patients are alive, and 2 have died. One melanoma patient died of pneumonia 30 months post‐TA, having developed multiple site metastases during the follow‐up period. The second patient treated for a solitary ENT metastasis and developed mediastinal lymph nodes metastases and a large pancreatic lesion (likely metastatic); he died of abdominal complications 37 months post TA. No local recurrence was noted in any patient. The average post‐procedural follow‐up for the eight patients was 40 months (ranging from 28‐60 months). We make note of one patient who developed a suspicious nodule outside of the treatment region, directly onto the Cyberknife fiducial, likely related to tumoral seeding (breast cancer metastasis). This patient opted for surgical lobectomy shortly after, and no local recurrence was recorded.

## 4 Discussion

TA of lung lesions has been performed since the 1990s with increasing rates of success as the inclusion criteria were refined [3]. Current guidelines for local treatment of lung lesions give preference to surgical treatment of lung lesions, and to a certain degree, SABRT is granted the benefit of a slight potential advantage over TA in terms of local recurrence and overall survival, especially for larger lesions [4]. However, significant limitations of these guidelines exist, namely, the absence of randomised controlled studies comparing the different treatment modalities, heterogeneity and small size of study groups, and potential selection bias for better outcomes with surgical and SABRT treatment in comparison with TA [1]. In addition, in the data sets studying thermal ablation results, noninferior outcomes were reported for nodules smaller than 2 cm, in terms of rates of overall survival and local control [5]. It is for these reasons that we have limited our “one stop shop” diagnosis‐treatment approach to small lung lesions that also had the benefit of a favourable technical approach. As such, larger, more central or directly subpleural lesions and lesions surrounded by gross emphysematous changes or in direct contact with significant vascular structures were avoided, in order to reduce the risk of complications (namely, pneumothorax and haemorrhage) that might lead to hospitalisation and prevent same day discharge and that are also more appropriately treated under general anaesthesia.

Large studies comparing the different TA modalities are not available. Limited data seem to support the idea that in terms of overall survival and local tumoral control, microwave ablation and cryoablation have similar outcomes, also comparable to the more traditional radiofrequency ablation literature [6]. During the current series, 6 treatments were carried out by microwave ablation and 2 by cryoablation; we tend to favour microwave ablation mainly due to its shorter treatment time (2–6 min versus 25–30 min). The standardised local pleural anaesthesia technique, described elsewhere [2], has enabled us to carry out all procedures without general anaesthesia, with only one patient requiring light sedation, mainly to alleviate anxiety. All patients reported minimal or no discomfort. While the microwave‐treated patients reported slightly more local pain, the two cryoablation‐treated patients were discomforted by the need to remain immobile for the longer duration of the procedure. For this reason, microwave ablation was generally preferred.

This brief report describes our experience with a rapid, same session diagnosis and treatment procedure of small neoplastic lesions that are relatively easy to access under CT guidance. A close collaborative interdisciplinary approach between radiation oncology, surgery, and radiology specialists allowed us to identify a set of patients for whom a same day diagnosis, treatment, and hospital discharge were deemed appropriate. Our initial experience shows an overall positive outcome: on‐site rapid cytological diagnosis was possible in all eight patients selected for the procedure; subsequent TA under local anaesthesia was successful in all cases; all patients were discharged the same day (only one pneumothorax required tube drainage, and the tube was subsequently removed after a short 4‐h observation period); no hospital admission was recorded within the 30 days following the intervention. We are aware, however, that despite the technical success of this small cohort, difficulties may arise in certain cases, such as an equivocal or non‐diagnostic cytological diagnosis. In this sort of instance, we might entertain the possibility of treating the lesion even in the absence of definite cytological proof of neoplasia, if the pre‐intervention imaging is highly suspicious; or perform a core biopsy, using a 20 G needle inserted through the 19 G coaxial, and wait for the pathological result before proceeding with the TA at a later date.

A rapid, same day diagnosis and treatment approach has multiple potential advantages. Among general situations, we would count less time away from work or home (and potentially from dependent family members or pets), less patient exposure to hospital environment (and potential nosocomial infections), reduced anxiety, and last but not least, a significant cost reduction, as compared with regular multi‐day evaluation, diagnosis, and treatment sequences as an inpatient or during multiple radiotherapy sessions. Local pleural anaesthesia, as opposed to general anaesthesia, is also an option more suitable for the patient population that we have selected, with a less likelihood of significant procedural complications. A same day diagnosis and treatment sequence was already described in the literature, utilising radiofrequency ablation [7]; to our knowledge, there are no reports of such an approach using microwave ablation or cryoablation. In our experience, the most convenient ablation method in the setting of easy access, small tumours, approached under local anaesthesia, is the microwave ablation, given its shorter treatment protocol.

This report has some limitations. The small number of patients precludes meaningful statistical analyses, for example, when analysing survival and local recurrence rates or when comparing microwave versus cryoablation treatment methods. In our experience, while perilesional haemorrhage sometimes led to less precise intraprocedural tumour localization, this did not affect the overall result (the surrounding anatomic landmarks—vessels and bronchi configuration—were sufficient for adequate probe positioning).

Further work with adequate statistical power is needed for definite validation of the rapid diagnosis, treatment, and hospital discharge sequence. Subsequently, the possibility of extending this approach to larger, more central, or subpleural lesions will also need to be explored. At a larger scale, despite the decade‐long experience with thermoablation documented in the literature, precise guidelines that integrate thermoablation along with SBRT and sublobar wedge resection in the flowcharts of lung cancer treatment do not yet exist, and further analysis, in particular randomised controlled trials, is needed in order to clarify the adequate role of each treatment modality.

## Author Contributions

**Andrei B. Gorgos:** conceptualization, investigation, methodology, validation, writing – review and editing, writing – original draft, supervision. **Damien Olivié:** design of study and/or critical review of paper. **Philippe Stephenson:** design of study and/or critical review of paper. **Edith Filion:** design of study and/or critical review of paper, clinical management of study participants and data acquisition. **Houda Bahig:** design of study and/or critical review of paper, clinical management of study participants and data acquisition.

## Conflicts of Interest

The authors declare no conflicts of interest.

## Data Availability

The data that support the findings of this study are available from the corresponding author upon reasonable request.
